# Dipstick proteinuria level is significantly associated with pre-morbid and in-hospital functional status among hospitalized older adults: a preliminary study

**DOI:** 10.1038/srep42030

**Published:** 2017-02-08

**Authors:** Chia-Ter Chao, Hung-Bin Tsai, Chih-Kang Chiang, Jenq-Wen Huang, Kuan-Yu Hung

**Affiliations:** 1 National Taiwan University College of Medicine, NO.1, Jen-Ai Road Section 1, Taipei 10051, Taiwan; 2 National Taiwan University Hospital, NO.7, Chung-Shan South Road, Zhong-Zheng district, Taipei 10002, Taiwan; 3 National Taiwan University Hospital Jin-Shan branch, NO.7, Yulu Road, Jin-Shan district, New Taipei City 208, Taiwan; 4 National Taiwan University Hospital Hsin-Chu branch, NO.25, Lane 442, Jingguo Road Section 1, Hsin-Chu City 300, Taiwan; 5 Department of Traumatology; National Taiwan University Hospital, Taipei, Taiwan; 6 Department of Integrative Diagnostics and Therapeutics, National Taiwan University Hospital, Taipei, Taiwan; 7 Department of Internal Medicine, National Taiwan University Hospital Hsin-Chu branch, Hsin-Chu County, Taiwan

## Abstract

Although chronic kidney disease (CKD) is associated with functional decline, whether proteinuria alone is associated with functional statuses over the course of acute illnesses independent of CKD is unclear. During 2014, we prospectively enrolled non-dialysis patients aged ≥65 years, and all participants underwent spot dipstick urinalysis on admission, divided into 3 groups according to the results (none, trace to 1 + , and 2 + or higher); functional status was evaluated using the pre-morbid and in-hospital Barthel index (BI) scores. Of 136 community-dwelling elderly patients enrolled (age 80.7 ± 8.2 years, with 19% having CKD), 17%, 57%, and 26% had no, trace to 1 + , or 2 + or higher proteinuria. Overall pre-morbid, on-admission, and on-discharge BI scores were 50.4 ± 41.9, 38.6 ± 31.8, and 38.7 ± 35.3, respectively with significant negative correlations with proteinuric severity on admission. Finally, multivariate linear stepwise regression analysis with backward variable selection found that dipstick proteinuric severity was significantly associated with pre-morbid, on-admission, and on-dischrage BI scores (*p* = 0.048, <0.01, and <0.01, respectively), independent of diabetes and CKD. This relationship between dipstick proteinuric levels and functional status of hospitalized elderly suggests an under-recognized association. Prospective evaluation of long-term outcome is needed.

Elderly are frequently hospitalized due to their age-associated organ degeneration, the presence of co-morbidities, and their susceptibility to adverse insults. Alterations in functional status often occur during hospitalization, and the degree of functional decline can parallel the severity of illnesses. For older persons, gauging their pre-morbid and in-hospital functional status facilitates treatment planning and potentially functional restoration^1^,^2^,^3^. While the identification of risk factors or markers of poor pre-morbid and in-hospital functional status may help facilitate this process, this area remains under-researched to date. Factors associated with functional decline in the hospitalized elderly include the types of morbidities and the reasons for their admission. Indeed, elderly with chronic kidney disease (CKD) are more likely to exhibit functional decline, beginning from the earlier stage of CKD to end-stage renal disease (ESRD)^4^,^5^; functional dependency also predisposes individuals with CKD and ESRD to recurrent hospitalization and higher mortality.

Albuminuria and proteinuria, as the staging criteria for CKD in the most recent version of Kidney Disease Improving Global Outcomes (KDIGO) CKD guidelines^6^, are both well-established predictors of subsequent renal function decline^7^. There is increasing awareness that albuminuria and proteinuria have an independent role in the prediction of adverse outcomes apart from the baseline renal function^8^. As explained above, although CKD is associated with poor functional status, it is still unclear whether proteinuria alone exhibits similar association with functional status regardless of CKD. No reports focus on this association using the severity of proteinuria among geriatric patients with acute medical illnesses.

We hypothesized that elderly with proteinuria on admission, regardless of the presence of CKD, are more likely to have poor functional status, and that a dose-responsive relationship between the severity of proteinuria and that of functional impairment exists. Therefore, we conducted a cross-sectional study to evaluate this theory.

## Materials and Methods

### Recruitment of participants and the study design

Elderly patients aged ≥65 years who were admitted to the general wards for medical illnesses between January and June 2014, were prospectively enrolled. Those who died within 2 days of admission, those who had anuria on presentation, or those who received chronic dialysis for ESRD were excluded. All participants underwent spot fasting dipstick urinalysis early on the day of admission, and were subsequently divided into 3 groups according to the semiquantitative levels of proteinuria assessed by dipstick (none, trace to 1+, and 2+ to higher). Demographic profile (age and sex), baseline comorbidity including hypertension, diabetes mellitus (DM), cardiovascular illnesses (coronary artery disease, myocardial infarction, heart failure, peripheral vascular disease, and old stroke), chronic obstructive pulmonary disease, CKD, autoimmune disorders, malignancy, peptic ulcer, and dementia/Parkinsonism were recorded based on patient history and corresponding laboratory and imaging findings. CKD was defined as an estimated glomerular filtration rate (eGFR) less than 60 ml/min/1.73 m^2^, using the Chronic Kidney Disease Epidemiology Collaboration (CKD-EPI) formula. The Charlson comorbidity index was calculated according to the literature^9^,^10^. Physical examination parameters including blood pressure and heart rate were documented. All participants received a hemogram and serum biochemical tests on admission.

### Functional status as the outcome of interest

Pre-morbid and in-hospital functional status among all the participants was evaluated using BI, through a standardized patient interview during admission conducted by dedicated nurse researchers. In brief, participants received BI assessment on the first day of their admission (the “on-admission scores”), and pre-morbid functional status was simultaneously recorded through asking the participants to recall their performance status one month prior to this admission (the “pre-morbid scores”). On the day of discharge, their BI scores were recorded again by the same group of interviewers (“the on-discharge scores”). Percentage changes between pre-morbid/on-admission and on-admission/on-discharge BI scores were also calculated.

BI is a classic tool for measuring performance in activities of daily living, and consists of 10 variables (15 points for 2 items: transfer from bed to chair, and walking; 10 points for 6 items: feeding, dressing, bowel and bladder continence, toileting, and stair-climbing; and 5 points for 2 items: bathing and grooming). The composite scores range between 0 and 100, with higher scores indicating better performance. Surrogate respondents (family members) were used for those who could not be interviewed due to severe cognitive impairment or an inability to communicate. Two interviews were done consecutively within one hour by different interviewers on the day of admission (for pre-morbid [by recall] and on-admission scores) or on the day of discharge (for on-discharge scores), so that the process of assessment would not disturb the routine admission care and the discharge preparation process. If there were discrepancies between the results obtained, we would average the two numbers and derive the final results.

The study protocol was approved by the Institutional Review Board of National Taiwan University Hospital (No. 201306089RINA), and adhered to the Declaration of Helsinki. All participants provided verbal informed consent before enrollment. For the analysis of factors associated with pre-morbid BI scores, only those with available pre-morbid BI scores were analyzed; for the analysis of factors associated with on-admission and on-discharge BI scores, data from all participants were used.

### Statistical analysis

To analyze the cross-sectional relationship between functional status at different scenarios and dipstick proteinuria results on admission, we first evaluated the differences among demographic profiles, comorbidities, vital sign parameters, and laboratory profiles between participants with and without proteinuria, using Student’s *t*-test, and between participants with different degrees of proteinuria, using Analysis of Variance (ANOVA) with Bonferroni correction. Results of BI subscales were also compared between participants with different degrees of proteinuria on admission, using ANOVA with Bonferroni correction as well. Finally, multivariate stepwise regression analyses with backward variable selection, with the BI scores in the form of continuous parameters as the dependent variable were performed, incorporating demographic profiles (age and gender), all comorbidities (diabetes, hypertension, cirrhosis, coronary artery disease, old infarction, peripheral vascular disease, chronic obstructive pulmonary disease, chronic kidney disease, malignancy, peptic ulcer, old stroke, and dementia or Parkinsonism), the Charlson comorbidity index, vital signs (blood pressure and heart rate), laboratory parameters, and dipstick proteinuria results. In this study, all analyses were done using SPSS 18.0 software (Chicago, IL, USA); a two-sided *p*-value less than 0.05 was considered statistically significant.

## Results

### Clinical features of the recruited patients

A total of 136 community-dwelling elderly patients (mean age 80.7 ± 8.2 years; 50% male) were enrolled during the study period. More than half of the participants had hypertension (57%), followed by DM (39%), malignancy (26%), and a prior stroke (21%), and CKD (19%). Among all participants, 17% did not have proteinuria, while 57% and 26% had trace to 1 + and 2 + to higher levels of proteinuria on admission, respectively. We did not observe any differences with regard to age and sex between patients with proteinuria and those without (Table 1). Proteinuric patients were more likely to have DM (*p* < 0.01), CKD (*p* = 0.01), and dementia/Parkinsonism (*p* = 0.03) than those without proteinuria, while those with higher severity of proteinuria also had higher prevalence of DM (*p* < 0.01) and CKD (*p* < 0.01) (Table 1). Charlson index scores were significantly higher among proteinuric patients than those without proteinuria (*p* < 0.01); Charlson index scores were also higher as the severity of proteinuria increased (*p* < 0.01). Finally, we observed no differences with regard to systolic, diastolic blood pressure, and heart rate between patients with proteinuria and those without. Proteinuric patients had a significantly higher leukocyte count (*p* = 0.04) and serum creatinine (*p* = 0.01) than those without proteinuria, while those with higher proteinuric severity also had higher leukocyte count (*p* < 0.01) and serum creatinine (*p* = 0.01) (Table 1).

### Functional status assessment results using BI

**Figure 1:**
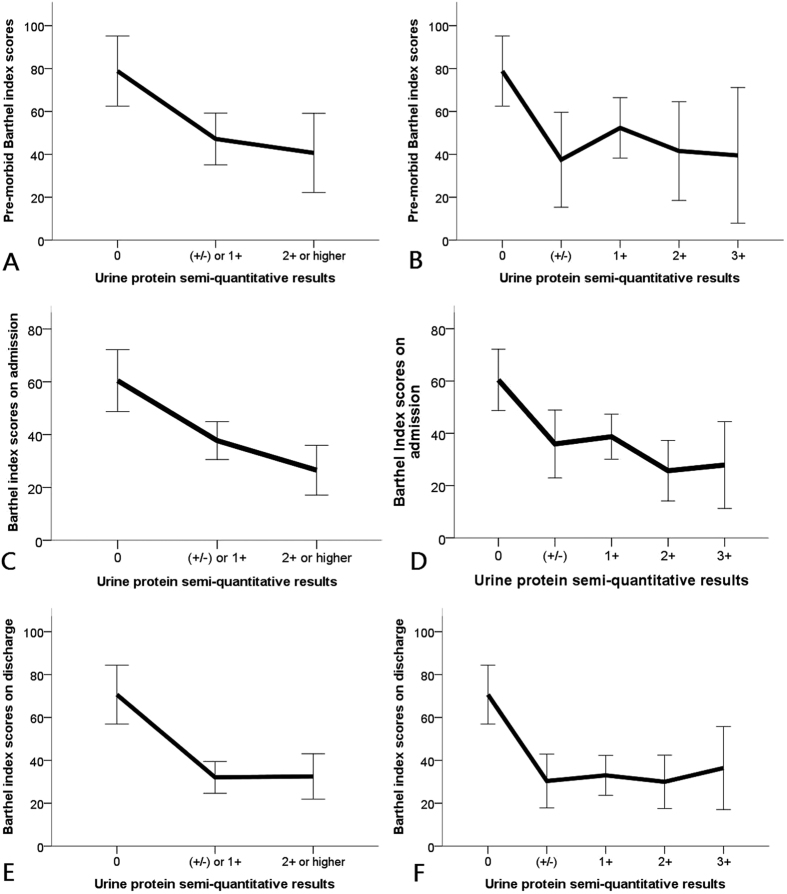
The left side panels illustrated the relationship between dipstick proteinuric levels and pre-morbid (**A**), on-admission (**C**), and on-discharge (**E**) functional status, as measured by Barthel index among hospitalized elderly participants, divided into 3 groups; the right side panels illustrated the relationship between dipstick proteinuric levels and pre-morbid (**B**), on-admission (**D**), and on-discharge (**F**) Barthel index scores among all participants, divided into 5 groups.

Pre-morbid BI scores were available among 82 (60.3%) participants, and there were no significant differences with regard to demographic profiles, any comorbidity, and other clinical features between those with and without pre-morbid data (supplementary Table). The average pre-morbid, on-admission, and on-discharge BI scores in this cohort were 50.4 ± 41.9, 38.6 ± 31.8, and 38.7 ± 35.3, respectively. We found that 50% participants had pre-morbid BI scores higher than 60, while 35.3% and 33.1% participants had on-admission and on-discharge BI scores higher than 60. Those with higher severity of proteinuria on admission also had significantly lower pre-morbid (for none, trace to 1 + , 2 + to higher, 78.9 ± 29.5, 47.2 ± 40.9, and 40.7 ± 44.4, respectively; *p* = 0.02), on-admission (for none, trace to 1 + , 2 + to higher, 60.4 ± 28.1, 37.7 ± 31.4, and 26.5 ± 28.3, respectively; *p* < 0.01), and on-discharge BI scores (for none, trace to 1 + , 2 + to higher, 70.7 ± 33, 32.1 ± 32.6, and 32.5 ± 31.8, respectively; *p* < 0.01) (Fig. 1). This inverse relationship between the severity of proteinuria and pre-morbid, on-admission, and on-discharge BI scores remained significant if proteinuria results were further divided into none, trace, 1 + , 2 + , and 3 + (*p* = 0.05, < 0.01, and < 0.01 for pre-morbid, on-admission, and on-discharge scores, respectively; Fig. 1).

On further analysis, we found that patients with dipstick proteinuria also had significantly lower pre-morbid BI subscale scores, including those for feeding (*p* = 0.04), grooming (*p* < 0.01), bathing (*p* = 0.02), bowel (*p* < 0.01) and bladder continence (*p* < 0.01), dressing (*p* < 0.01), walking (*p* = 0.02), and stair-climbing (*p* = 0.01) (Table 2). Similarly, patients with dipstick proteinuria also had significantly lower on-admission BI subscale scores, including those for feeding (*p* = 0.01), grooming (*p* < 0.01), bowel (*p* < 0.01) and bladder continence (*p* < 0.01), toileting (*p* < 0.01), dressing (*p* < 0.01), transfer (*p* < 0.01), walking (*p* < 0.01), and stair-climbing (*p* < 0.01) (Table 3). Patients with dipstick proteinuria also had significantly lower on-discharge BI subscale scores, including those for feeding (*p* < 0.01), grooming (*p* < 0.01), bathing (*p* < 0.01), bowel (*p* < 0.01) and bladder continence (*p* < 0.01), toileting (*p* < 0.01), dressing (*p* < 0.01), transfer (*p* < 0.01), walking (*p* < 0.01), and stair-climbing (*p* < 0.01) (Table 4). Higher severity of proteinuria was also associated with lower BI subscale scores in most scenarios.

Among the participants, the percentage change between pre-morbid and on-admission BI scores was 43.2 ± 35.9%, while that between on-admission and on-discharge BI scores was 45.2 ± 50.1%. Participants with and without proteinuria had similar percentage changes between pre-morbid/on-admission (proteinuric vs. non-proteinuric, 45.1 ± 36.2% vs. 34 ± 35%, *p* = 0.36) and on-admission/on-discharge BI scores (48.5 ± 53.3% vs. 28.8 ± 24.8%, *p* = 0.09). In addition, if dipstick results were divided into none, trace to 1 + , and 2 + to higher, no significant differences were observed among those with different proteinuric severity, with regard to percentage changes between pre-morbid/on-admission BI scores (none vs. trace to 1 + vs. 2 + to higher, 34 ± 35% vs. 43.9 ± 35.4% vs. 47.4 ± 38.4%, *p* = 0.62), or to those between on-admission/on-discharge BI scores (none vs. trace to 1 + vs. 2 + to higher, 28.8 ± 24.8% vs. 48.4 ± 51.4% vs. 48.9 ± 58%, *p* = 0.23). Similarly, if dipstick results were divided into none, trace, 1 + , 2 + , or 3 + , no significant differences were observed with regard to percentage changes between pre-morbid/on-admission BI scores (none vs. trace vs. 1 + vs. 2 + vs. 3 + , 34 ± 35% vs. 52.1 ± 44.4% vs. 40.8 ± 31.9% vs. 41.6 ± 38.7% vs. 53.8 ± 39.3%, *p* = 0.7), or to those between on-admission/on-discharge BI scores (none vs. trace vs. 1 + vs. 2 + vs. 3 + , 28.8 ± 24.8% vs. 38.1 ± 44.2% vs. 54 ± 54.5% vs. 45.8 ± 59.9% vs. 53.7 ± 56.6%, *p* = 0.3).

### Regression analyses assessing the relationship between the severity of proteinuria and BI scores during the course of acute medical illnesses

Finally, we conducted multivariate stepwise regression analyses with backward variable selection, to evaluate the relationship between dipstick proteinuric severity and pre-morbid, on-admission, and on-discharge functional status. After accounting for demographic variables (age and gender), all comorbidities, and laboratory parameters (leukocyte counts, hemoglobin, platelet counts), linear regression analyses with BI scores as the dependent variable showed that participants with higher degree of proteinuria had significantly poorer pre-morbid (*p* = 0.048), on-admission (*p* < 0.01), and on-discharge functional status (*p* < 0.01) (Table 5). This relationship was independent of baseline DM or CKD status. A sensitivity analysis using an alternative categorization (none, trace, 1 + , 2 + , and 3 + ) similarly showed that the severity of proteinuria was significantly associated with BI score at different scenarios (for on-admission scores, *t* = −3.1, β = −0.24, *p* < 0.01; for on-discharge scores, *t* = −2.21, β = −0.17, *p* = 0.03). Sensitivity analyses accounting for the presence of acute kidney injury diagnosis on admission, or focusing solely on non-diabetic, non-hypertensive, or non-CKD participants yielded essentially similar results (Table 5).

## Discussion

In the current study, we found that among a cohort of prospectively enrolled elderly patients admitted for medical illnesses, those with dipstick proteinuria had significantly lower pre-morbid, on-admission, and on-discharge BI scores. Those with a higher degree of proteinuria on admission were more likely to have worse BI scores, and this relationship applied to most subscales of BI. Finally, regression models accounting for demographic profiles, comorbidities, and laboratory data showed that the relationship between proteinuric severity and functional status was independent of age, the presence of DM, CKD, old stroke, and serum creatinine on admission. It is therefore likely that the level of dipstick proteinuric severity can be closely associated with functional status, an important outcome-determining factor for acutely hospitalized elderly patients.

The prevalence of proteinuria (from trace to 2 + and higher) in our study was higher than that reported by others. Population surveys reported that about one-fourth of septuagenarians manifest microalbuminuria^11^. Our enrollees had an advanced average age, and around 57% had hypertension, while only 39% and 19% had DM and CKD, respectively. Thus, age-related vascular aging and hypertension-related microalbuminuria might be partially responsible for the higher prevalence of dipstick proteinuria in our study. In addition, the acute illness prompting admission might also play a role in the prevalence of proteinuria on admission. Proteinuria can be a physiologic response to external stressors, such as fever and sepsis, which are common scenarios in acutely hospitalized patients, by affecting renal vascular tone^12^. Furthermore, about 40% of our enrolled participants were admitted for community-acquired pneumonia; evidence suggests that bacterial pneumonia, particularly pneumococcal pneumonia, is also associated with the appearance of proteinuria^13^,^14^. Nonetheless, sensitivity analyses revealed that the independent relationship between proteinuric severity and BI scores exists even among those without hypertension or DM, and the relationship was borderline significant or those without CKD (Table 5). These findings collectively indicate that these comorbidities might not significantly affect our results.

Common approaches for detecting proteinuria include the direct measurement of the urinary protein to creatinine ratio and the urine dipstick strip for semiquantitative measurement of protein. Previous studies have questioned the utility of dipstick proteinuria to predict the actual severity measured by direct protein quantification, especially when the proteinuria is severe or when screening for microalbuminuria is desired^15^. Despite its lower sensitivity for microalbuminuria and the potential for underestimating proteinuric severity, dipstick proteinuria still exhibits a strong and graded association with the risk for ESRD and mortality in the general population and those with CKD^16^,^17^. In addition, there are reports suggesting that the severity of albuminuria correlated strongly with that of proteinuria, especially among elderly patients, the target population of this study^18^. Since the direct measurement of urinary albumin or protein is more expensive than a urine dipstick test and takes longer to obtain results, a urine dipstick for proteinuria might be economically preferable. Furthermore, we discovered an under-recognized but close relationship between dipstick proteinuria and not only pre-morbid functional status, but also that during admission and on discharge in the hospitalized elderly, further lending support to the importance of measuring dipstick proteinuria.

Existing literature rarely addresses the relationship between proteinuria and functional status. Turaj *et al*. reported that non-diabetic stroke patients with microalbuminuria might score lower on the BI, and another group reported similar results in post-stroke patients receiving neurologic rehabilitation^19^,^20^. Ovbiagele *et al*. verified these results in stroke patients without known renal disease^21^. We further extended their findings by showing that the relationship between proteinuria and functional status was dose-dependent (Fig. 1), independent of CKD status, and was applicable to geriatric patients with or without stroke. There are several factors that could explain our findings. Microalbuminuria is reportedly associated with salt sensitivity, chronic inflammation, and insulin resistance, all of which contribute to endothelial dysfunction and vasculopathy development^10^. Microalbuminuria has also been recognized as a subclinical marker for atherosclerosis and microvascular diseases, representing a state of compromised functional or structural integrity of the vasculature^20^,^22^,^23^. Consequently, escalating severity of proteinuria could be a surrogate for more severe advanced cerebral small vessel disease and lacunar strokes, contributing to subsequent functional decline over time^24^. Heavy proteinuria by dipstick testing also predicts a higher risk of peripheral vascular disease, which has been shown to negatively affect activities of daily living^25^. Proteinuria might directly lead to lower functional status by contributing to hypercoagulability and the development of ischemic stroke and/or lower extremity venous thromboembolism^26^. Finally, proteinuria might serve as a marker for the overall severity of illness on admission, and is inversely associated with functional status after discharge^27^,^28^.

Past reports indicate that diseases with higher severity lead to more prominent functional decline^29^. Our findings suggest that dipstick proteinuria might be a token of the combination of an individual’s functional status before an acute illness occurs and that during the acute illness as well. More importantly, there was no obvious association between the severity of proteinuria and functional decline observed during admission, further exemplifying our proposition that the independent relationship between proteinuria and functional status is present regardless of the acute illness *per se*. Functional status, presenting as BI scores, and proteinuria might share other pathophysiologic connections, as suggested above.

Our study has several limitations. The number of enrolled cases is not large, which might limit the applicability of our findings. This may also explain the borderline significant findings in our sensitivity analysis focusing on non-CKD participants. In addition, pre-morbid BI scores were available in about 60% participants, significantly limiting the generalizability of our findings to other elderly. However, if results with statistical significance can be discovered after analyzing the current cohort, we believe that the relationship between functional status and the severity of proteinuria we observed truly exists. Studies using larger cohort are needed for the validation of our findings. The use of BI alone might not fully capture the spectrum of functional status assessment for these patients, but the repeated verification of individual BI scores in this study and the uniform and standardized urine collection procedures increases the credibility of our results. Judging from the close relationship between the severity of proteinuria and pre-morbid, on-admission, and on-discharge functional status we observed, it would be prudent to pay more attention to older in-patients with severe proteinuria found during admission, as these patients are at higher risk of manifesting poorer function status even at discharge. Finally, unmeasured confounding factors of pre-morbid status and during admission might exist in this study, limiting the interpretation of our results.

## Conclusion

We found a close relationship between dipstick proteinuria severity and functional status before, during, and after acute medical illnesses among older adults. Although measuring dipstick proteinuria levels cannot replace the practice of geriatric functional assessment, positive dipstick proteinuria still serves to alert us that the patient can be at higher risk of having poorer function before and after the acute insults. Formal functional status evaluation is recommended for these patients, in order to facilitate subsequent treatment planning for functional recovery.

## Additional Information

**How to cite this article:** Chao, C.-T. *et al*. Dipstick proteinuria level is significantly associated with pre-morbid and in-hospital functional status among hospitalized older adults: a preliminary study. *Sci. Rep.* **7**, 42030; doi: 10.1038/srep42030 (2017).

**Publisher's note:** Springer Nature remains neutral with regard to jurisdictional claims in published maps and institutional affiliations.

## Supplementary Material

Supplementary Table

## Figures and Tables

**Table 1:** Clinical features of the elderly participants.

Clinical features	Negative (n = 23)	Trace to 1+ (n = 77)	2+ to higher (n = 36)	*P1 value*	Positive (n = 113)	*P2 value*
***Demographic profiles***						
Age (years)	79.1 ± 9.9	81.3 ± 8.1	80.4 ± 7.2	*0.52*	80.1 ± 7.8	*0.31*
Gender (male %)	10 (43)	38 (49)	20 (56)	*0.66*	58 (51)	*0.5*
***Comorbidities (%)***						
Diabetes mellitus	1 (4)	33 (43)	19 (53)	*<0.01*	52 (46)	*<0.01*
Hypertension	10 (43)	47 (61)	21 (58)	*0.33*	68 (60)	*0.14*
Cirrhosis	2 (9)	4 (5)	2 (6)	*0.82*	6 (5)	*0.53*
Coronary artery disease	2 (9)	3 (4)	6 (17)	*0.07*	9 (8)	*0.91*
Old myocardial infarction	0 (0)	1 (1)	0 (0)	*0.69*	1 (1)	*0.65*
Heart failure	2 (9)	16 (21)	5 (14)	*0.35*	21 (19)	*0.25*
Peripheral vascular disease	0 (0)	9 (12)	0 (0)	*0.02*	9 (8)	*0.16*
Chronic obstructive pulmonary disease	3 (13)	8 (10)	4 (11)	*0.94*	12 (11)	*0.74*
Chronic kidney disease	0 (0)	13 (17)	13 (36)	*<0.01*	26 (23)	*0.01*
Rheumatologic disorders	0 (0)	4 (5)	0 (0)	*0.21*	4 (4)	*0.36*
Malignancy	5 (22)	21 (27)	9 (25)	*0.86*	30 (27)	*0.63*
Peptic ulcer disease	2 (9)	4 (5)	6 (17)	*0.14*	10 (9)	*0.98*
Old stroke	4 (17)	18 (23)	6 (17)	*0.66*	24 (21)	*0.68*
Dementia or Parkinsonism	0 (0)	15 (19)	4 (11)	*0.06*	19 (17)	*0.03*
*Charlson Comorbidity Index*	6.2 ± 1.9	7.9 ± 2.4	8 ± 2.2	*<0.01*	7.9 ± 2.4	*<0.01*
***Vital signs on admission***						
Systolic blood pressure (mmHg)	141.7 ± 44.8	136.4 ± 36.1	130 ± 39.7	*0.49*	134.3 ± 37.2	*0.41*
Diastolic blood pressure (mmHg)	80.1 ± 24.3	74 ± 19.6	72.7 ± 20.8	*0.37*	73.6 ± 19.9	*0.17*
Heart rate (/minute)	93.7 ± 16.8	98.9 ± 21.2	93.9 ± 22.3	*0.38*	97.3 ± 21.6	*0.45*
***Laboratory parameters on admission***						
Leukocyte count (K/μL)	9.8 ± 6.2	11.7 ± 5.8	15.1 ± 6.7	*<0.01*	12.8 ± 6.3	*0.04*
Hemoglobin (mg/dL)	11.2 ± 2.5	12.6 ± 11.5	10.5 ± 2.7	*0.49*	11.9 ± 9.6	*0.74*
Platelet count (K/μL)	228 ± 117	232 ± 95	208 ± 110	*0.51*	225 ± 100	*0.88*
Serum creatinine (mg/dL)	0.8 ± 0.3	1.5 ± 1.5	2.3 ± 1.8	*0.01*	1.8 ± 1.6	*0.01*
Serum sodium (meq/L)	134 ± 4	132 ± 6.9	134 ± 7.8	*0.36*	133 ± 7.2	*0.59*
Serum potassium (meq/L)	4.6 ± 2.1	4.5 ± 1.1	4.6 ± 1	*0.09*	4.5 ± 1.1	*0.33*

Data are expressed as mean ± standard deviation for continuous variables, and number (percentage) for categorical variables.

*P1*: comparison between participants with different severity of proteinuria.

*P2*: comparison between participants with and without proteinuria.

**Table 2:** Details of pre-morbid Barthel index subscales among participants with different proteinuric severity.

Barthel index subscales	Negative (n = 13)	Trace to 1+ (n = 46)	2+ to higher (n = 23)	*P1 value*	Positive (n = 69)	*P2 value*
Feeding	8.5 ± 3.2	6 ± 4.5	5 ± 4.8	*0.08*	5.7 ± 4.6	*0.04*
Grooming	4.2 ± 1.9	2.3 ± 2.5	2 ± 2.5	*0.02*	2.2 ± 2.5	* < 0.01*
Bathing	3.1 ± 2.5	1.6 ± 2.4	1.5 ± 2.4	*0.07*	1.6 ± 2.3	*0.02*
Bowel continence	8.8 ± 3	4.9 ± 4.7	4.8 ± 5.1	*0.02*	4.9 ± 4.8	* < 0.01*
Bladder continence	8.5 ± 3.2	4.3 ± 4.4	3.5 ± 4.4	*<0.01*	4.1 ± 4.4	*<0.01*
Toileting	8.1 ± 3.3	4.3 ± 4.5	3.7 ± 4.8	*0.35*	4.1 ± 4.6	*0.47*
Dressing	8.1 ± 3.8	4.6 ± 4.3	3.7 ± 4.3	*0.01*	4.3 ± 4.3	*<0.01*
Transfer from bed to chair	11.5 ± 5.2	8.5 ± 6.5	6.7 ± 7.2	*0.11*	7.9 ± 6.7	*0.07*
Walking on level surface	11.2 ± 5.5	6.8 ± 6.9	5.4 ± 6.2	*0.04*	6.4 ± 6.7	*0.02*
Stair climbing	6.9 ± 4.3	3.8 ± 4.5	3 ± 4.2	*0.04*	3.6 ± 4.4	*0.01*

*P1*: comparison between participants with different severity of proteinuria.

*P2*: comparison between participants with and without proteinuria.

**Table 3:** Details of on-admission Barthel index subscales among participants with different proteinuric severity.

Barthel index subscales	Negative (n = 23)	Trace to 1+ (n = 77)	2+ to higher (n = 36)	*P1 value*	Positive (n = 113)	*P2 value*
Feeding	6.8 ± 3.7	4.4 ± 4	4.2 ± 3.2	*0.04*	4.3 ± 3.8	*0.01*
Grooming	2.3 ± 2.6	1 ± 2	0.5 ± 1.5	*0.01*	0.8 ± 1.9	*<0.01*
Bathing	1.3 ± 2.2	0.7 ± 1.9	0.5 ± 1.5	*0.37*	0.6 ± 1.8	*0.17*
Bowel continence	8.8 ± 2.8	6 ± 4.3	6 ± 4.4	*0.03*	6 ± 4.3	*<0.01*
Bladder continence	8.5 ± 2.9	6.2 ± 4.3	4.5 ± 4.8	*<0.01*	5.6 ± 4.5	*<0.01*
Toileting	6 ± 3.5	3.6 ± 3.3	3.5 ± 3.3	*0.02*	3.6 ± 3.3	*<0.01*
Dressing	6.5 ± 3.3	3.6 ± 3.6	3.2 ± 3.1	*<0.01*	3.4 ± 3.4	*<0.01*
Transfer from bed to chair	9 ± 5.8	5.2 ± 5.2	4.8 ± 4.8	*0.01*	5.1 ± 5.1	*<0.01*
Walking on level surface	7.8 ± 5	4.6 ± 5.5	3 ± 4.7	*<0.01*	4.1 ± 5.2	*<0.01*
Stair climbing	4.8 ± 3.4	2 ± 2.5	1.3 ± 2.2	*<0.01*	1.8 ± 2.4	*<0.01*

*P1*: comparison between participants with different severity of proteinuria.

*P2*: comparison between participants with and without proteinuria.

**Table 4:** Details of on-discharge Barthel index subscales among participants with different proteinuric severity.

Barthel index subscales	Negative (n = 23)	Trace to 1+ (n = 77)	2+ to higher (n = 36)	*P1 value*	Positive (n = 113)	*P2 value*
Feeding	7.9 ± 3.5	3.4 ± 3.7	4 ± 4.2	*<0.01*	3.6 ± 3.9	*<0.01*
Grooming	2.9 ± 2.5	0.7 ± 1.8	0.9 ± 1.9	*<0.01*	0.8 ± 1.8	*<0.01*
Bathing	2.1 ± 2.5	0.4 ± 1.3	0.6 ± 1.6	*<0.01*	0.4 ± 1.4	*<0.01*
Bowel continence	9.2 ± 2.5	4.5 ± 4.4	5.4 ± 4.3	*<0.01*	4.8 ± 4.4	*<0.01*
Bladder continence	9.2 ± 2.5	4.3 ± 4.4	4.7 ± 5	*<0.01*	4.5 ± 4.6	*<0.01*
Toileting	7.1 ± 3.5	3 ± 3.5	3 ± 3.5	*<0.01*	3 ± 3.5	*<0.01*
Dressing	7.4 ± 3.5	2.8 ± 3.4	2.9 ± 3.5	*<0.01*	2.8 ± 3.4	*<0.01*
Transfer from bed to chair	11.6 ± 4.7	4.6 ± 5.4	5.3 ± 5.5	*<0.01*	4.8 ± 5.4	*<0.01*
Walking on level surface	11.6 ± 4.7	4.1 ± 5.4	4.1 ± 5.5	*<0.01*	4.1 ± 5.4	*<0.01*
Stair climbing	6.1 ± 3.6	1.9 ± 3.1	1.9 ± 3.2	*<0.01*	1.9 ± 3.1	*<0.01*

*P1*: comparison between participants with different severity of proteinuria.

*P2*: comparison between participants with and without proteinuria.

**Table 5:** Results from multivariate regression analyses, with pre-morbid, on-admission, or on-discharge BI scores as the dependent variables.

Results	*t* value	β coefficient	*p* value
***All participants***			
*Model 1 – Pre-morbid BI scores (n = 82)*			
Hypertension	2.03	0.24	0.047
Old stroke	−2.15	−0.32	0.04
Proteinuria on dipstick tests	−2.02	−0.27	0.048
*Model 2 – On-admission BI scores (n = 136)*			
Old stroke	−3.44	−0.26	<0.01
Dementia or Parkinsonism	−4.76	−0.37	<0.01
Proteinuria on dipstick tests	−3.85	−0.29	<0.01
*Model 3 – On-discharge BI scores (n = 136)*			
Malignancy	−3.21	−0.26	<0.01
Old stroke	−2.66	−0.21	<0.01
Dementia or Parkinsonism	−3.14	−0.25	<0.01
Proteinuria on dipstick tests	−3.62	−0.28	<0.01
***Non-DM participants***			
*Model 4 – Pre-morbid BI scores (n = 82)*			
Hypertension	2.86	0.35	<0.01
Old stroke	−2.29	−0.28	0.03
Proteinuria on dipstick tests	−2.9	−0.36	<0.01
*Model 5 – On-admission BI scores (n = 136)*			
Malignancy	−3.06	−0.29	<0.01
Old stroke	−2.45	−0.23	0.02
Dementia or Parkinsonism	−3.62	−0.34	<0.01
Proteinuria on dipstick tests	−3.34	−0.32	<0.001
*Model 6 – On-discharge BI scores (n = 136)*			
Malignancy	−2.42	−0.24	0.02
Peptic ulcer	2.63	0.26	0.01
Dementia or Parkinsonism	−3.15	−0.3	<0.01
Proteinuria on dipstick tests	−3.03	−0.3	<0.01
***Non-hypertensive participants***			
*Model 7 – Pre-morbid BI scores (n = 82)*			
Proteinuria on dipstick tests	−2.08	−0.36	0.047
*Model 8 – On-admission BI scores (n = 136)*			
Old stroke	−2.07	−0.24	0.04
Proteinuria on dipstick tests	−2.66	−0.32	0.01
*Model 9 – On-discharge BI scores (n = 136)*			
COPD	2.76	0.3	0.01
Charlson index scores	−3.01	−0.34	<0.01
Proteinuria on dipstick tests	−2.51	−0.28	0.02
***Non-CKD participants***			
*Model 10 – Pre-morbid BI scores (n = 82)*			
Hypertension	2.45	0.28	0.02
Old stroke	−2.44	−0.28	0.02
Proteinuria on dipstick tests	−1.83	−0.21	0.07
*Model 11 – On-admission BI scores (n = 136)*			
Malignancy	−2.03	−0.17	0.045
Old stroke	−2.89	−0.24	<0.01
Dementia or Parkinsonism	−3.96	−0.34	<0.01
Proteinuria on dipstick tests	−3.37	−0.28	<0.01
*Model 12 – On-discharge BI scores (n = 136)*			
Age	−2.02	−0.18	0.046
Malignancy	−2.91	−0.26	<0.01
Peptic ulcer	2.07	0.19	0.04
Dementia or Parkinsonism	−2.74	−0.24	<0.01
Proteinuria on dipstick tests	−2.7	−0.24	<0.01

Variables in all models included age, gender, all comorbidities, Charlson index, and vital signs, and laboratory profiles.

BI, Barthel index; CKD, chronic kidney disease; COPD, chronic obstructive pulmonary disease; DM, diabetes mellitus.

## References

[b1] Chao C. T. . The severity of initial acute kidney injury at admission of geriatric patients significantly correlates with subsequent in-hospital complications. Sci Rep. 5, 13925 (2015). 26355041 10.1038/srep13925 PMC4564739

[b2] Bootsma A. M., Buurman B. M., Geerlings S. E. & de Rooij S. E. Urinary incontinence and indwelling urinary catheters in acutely admitted elderly patients: relationship with mortality, institutionalization, and functional decline. J. Am. Med. Dir. Assoc. 14147, e7–e12 (2013). 10.1016/j.jamda.2012.11.002 23206725

[b3] Socorro Garcia A., de la Puente M., Perdomo B., Lopez Pardo P. & Baztan J. J. Functional status and mortality at month and year in nonagenarians hospitalized due to acute medical illness. Eur. J. Intern. Med. 26, 705–708 (2015). 26320014 10.1016/j.ejim.2015.08.007

[b4] Bowling C. B., Sawyer P., Campbell R. C., Ahmed A. & Allman R. M. Impact of Chronic Kidney Disease on Activities of Daily Living in Community-Dwelling Older Adults. J. Gerontol. Biol. Sci. Med. Sci. 66A, 689–694 (2011). 10.1093/gerona/glr043 PMC3110910 21459762

[b5] Chao C. T. . Cross-sectional study of the association between functional status and acute kidney injury in geriatric patients. BMC. Nephrol. 16, 186 (2015). 26552371 10.1186/s12882-015-0181-7 PMC4640369

[b6] Chapter 1: Definition and classification of CKD. Kidney. Int. Suppl. 3, 19–62 (2013). 10.1038/kisup.2012.64 PMC4089693 25018975

[b7] Hirayama A. . Blood Pressure, Proteinuria, and Renal Function Decline: Associations in a Large Community-Based Population. Am. J. Hypertens. 28, 1150–1156 (2015). 25673040 10.1093/ajh/hpv003

[b8] Chronic Kidney Disease Prognosis Consortium. Association of estimated glomerular filtration rate and albuminuria with all-cause and cardiovascular mortality in general population cohorts: a collaborative meta-analysis. Lancet. 375, 2073–2081 (2010). 20483451 10.1016/S0140-6736(10)60674-5 PMC3993088

[b9] Chao C. T. . The Impact of Dialysis-Requiring Acute Kidney Injury on Long-Term Prognosis of Patients Requiring Prolonged Mechanical Ventilation: Nationwide Population-Based Study. PLoS. ONE. 7, e50675 (2012). 23251377 10.1371/journal.pone.0050675 PMC3520952

[b10] Chao C. T. . Advanced age affects the outcome-predictive power of RIFLE classification in geriatric patients with acute kidney injury. Kidney. Int. 82, 920–927 (2012). 22763817 10.1038/ki.2012.237

[b11] Abdelhafiz A. H., Ahmed S. & El Nahas M. Microalbuminuria: Marker or Maker of Cardiovascular Disease. Nephron. Exp. Nephrol. 119 Suppl 1, e6–e10 (2011). 21832857 10.1159/000328015

[b12] Danziger J. Importance of Low-Grade Albuminuria. Mayo. Clin. Proc. 83, 806–812 (2008). 18613997 10.4065/83.7.806

[b13] Huang S. T. . Pneumococcal pneumonia infection is associated with end-stage renal disease in adult hospitalized patients. Kidney. Int. 86, 1023–1030 (2014). 24694991 10.1038/ki.2014.79

[b14] Beović B. . Aetiology and Clinical Presentation of Mild Community-Acquired Bacterial Pneumonia. Eur. J. Clin. Microbiol. Infect. Dis. 22, 584–591 (2003). 13680399 10.1007/s10096-003-0997-0

[b15] Meyer N., Mercer B., Friedman S. & Sibai B. M. Urinary dipstick protein: a poor predictor of absent or severe proteinuria. Am. J. Obstet. Gynecol. 170, 137–141 (1994). 8296815 10.1016/s0002-9378(94)70398-1

[b16] Tanihara S. . Proteinuria is a Prognostic Marker for Cardiovascular Mortality: NIPPON DATA 80, 1980-1999. J. Epidemiol. 15, 146–153 (2005). 16141633 10.2188/jea.15.146 PMC7851071

[b17] Iseki K., Ikemiya Y., Iseki C. & Takishita S. P roteinuria and the risk of developing end-stage renal disease. Kidney. Int. 63, 1468–1474 (2003). 10.1046/j.1523-1755.2003.00868.x 12631363

[b18] Turaj W. . The prognostic significance of microalbuminuria in non-diabetic acute stroke patients. Med. Sci. Monit. 7, 989–994 (2001). 11535947

[b19] Atkins R. C., Briganti E. M., Zimmet P. Z. & Chadban S. J. Association between albuminuria and proteinuria in the general population: the AusDiab Study. Nephrol. Dial. Transplant. 18, 2170–2174 (2003). 13679498 10.1093/ndt/gfg314

[b20] Sander D. . Microalbuminuria indicates long-term vascular risk in patients after acute stroke undergoing in-patient rehabilitation. BMC. Neurol. 12, 102 (2012). 23007013 10.1186/1471-2377-12-102 PMC3517490

[b21] Ovbiagele B. . Indices of Kidney Dysfunction and Discharge Outcomes in Hospitalized Stroke Patients without Known Renal Disease. Cerebrovasc. Dis. 28, 582–588 (2009). 19844098 10.1159/000247602

[b22] Ravera M. . Microalbuminuria and subclinical cerebrovascular damage in essential hypertension. J. Nephrol. 15, 519–524 (2002). 12455718

[b23] Böhm M. . Atrial fibrillation and heart rate independently correlate to microalbuminuria in hypertensive patients. Eur. Heart. J. 30, 1364–1371 (2009). 19383737 10.1093/eurheartj/ehp124

[b24] Makin S. D., Cook F. A., Dennis M. S. & Wardlaw J. M. Cerebral Small Vessel Disease and Renal Function: Systematic Review and Meta-Analysis. Cerebrovasc. Dis. 39, 39–52 (2015). 25547195 10.1159/000369777 PMC4335630

[b25] Mohler E. R. . Progression of asymptomatic peripheral artery disease over 1 year. Vasc. Med. 17, 10–16 (2012). 22363014 10.1177/1358863X11431106

[b26] van Schouwenburg I. M. . Elevated albuminuria associated with increased risk of recurrent venous thromboembolism: results of a population-based cohort study. Br. J. Haematol. 156, 667–671 (2012). 22233096 10.1111/j.1365-2141.2011.09018.x

[b27] Chan Carusone S. B., Walter S. D., Brazil K. & Loeb M. B. Pneumonia and Lower Respiratory Infections in Nursing Home Residents: Predictors of Hospitalization and Mortality. J. Am. Geriatr. Soc. 55, 414–419 (2007). 17341245 10.1111/j.1532-5415.2007.01070.x

[b28] Vogel T. R., Petroski G. F. & Kruse R. L. Functional status of elderly adults before and after interventions for critical limb ischemia. J. Vasc. Surg. 59, 350–358 (2014). 24139567 10.1016/j.jvs.2013.08.087 PMC4160828

[b29] Covinsky K. E., Pierluissi E. & Johnston C. B. Hospitalization-associated disability: “She was probably able to ambulate, but I’m not sure”. JAMA. 306, 1782–1793 (2011). 22028354 10.1001/jama.2011.1556

