# Supplementary material for: Dipstick proteinuria level is significantly associated with pre-morbid and in-hospital functional status among hospitalized older adults: a preliminary study
Source: Sci Rep. 2017 Feb 8;7:42030. doi: 10.1038/srep42030 (PMC5296719; doi:10.1038/srep42030)
Supplement: Supplementary Table [file srep42030-s1.doc]

***Dipstick proteinuria level is significantly associated with pre-morbid and in-hospital functional status among hospitalized older adults: a preliminary study***

Chia-Ter Chao, Hung-Bin Tsai, Chih-Kang Chiang, Jenq-Wen Huang, Kuan-Yu Hung

**Supplementary Table.** Clinical features of the elderly participants with and without pre-morbid Barthel index scores

| **Clinical features** | **Available pre-morbid scores (n = 82)** | **Unavailable pre-morbid scores (n = 54)** | ***P value*** |
| --- | --- | --- | --- |
| Demographic profile |  |  |  |
| Age (years) | 80.8 ± 8.5 | 80.5 ± 7.6 | 0.81 |
| Gender (male %) | 42 (51) | 26 (48) | 0.73 |
| *Comorbidities (%)* |  |  |  |
| Diabetes mellitus | 31 (38) | 22 (41) | 0.73 |
| Hypertension | 45 (55) | 33 (61) | 0.48 |
| Cirrhosis | 4 (5) | 4 (7) | 0.54 |
| Coronary artery disease | 6 (7) | 5 (9) | 0.69 |
| Old myocardial infarction | 1 (1) | 0 (0) | 0.42 |
| Heart failure | 17 (21) | 6 (11) | 0.15 |
| Peripheral vascular disease | 6 (7) | 3 (6) | 0.69 |
| Chronic obstructive pulmonary disease | 11 (13) | 4 (7) | 0.28 |
| Chronic kidney disease | 17 (21) | 9 (17) | 0.56 |
| Rheumatologic disorders | 4 (5) | 0 (0) | 0.1 |
| Malignancy | 21 (26) | 14 (26) | 0.97 |
| Peptic ulcer disease | 6 (7) | 6 (11) | 0.45 |
| Old stroke | 19 (23) | 9 (17) | 0.36 |
| Dementia or Parkinsonism | 10 (12) | 9 (17) | 0.47 |
| *Charlson Comorbidity Index* | 7.6 ± 2.3 | 7.8 ± 2.4 | 0.61 |
| *Vital signs on admission* | | | |
| Systolic blood pressure (mmHg) | 134.1 ± 32.8 | 137.7 ± 46.2 | 0.62 |
| Diastolic blood pressure (mmHg) | 75.9 ± 18.2 | 72.9 ± 24.2 | 0.41 |
| Heart rate (/minute) | 95.7 ± 19.7 | 98.2 ± 22.6 | 0.5 |
| *Laboratory parameters on admission* | | | |
| Leukocyte count (K/µL) | 12.4 ± 11.2 | 12.2 ± 6.3 | 0.88 |
| Hemoglobin (mg/dL) | 12.4 ± 11.2 | 10.8 ± 2.4 | 0.33 |
| Platelet count (K/µL) | 231 ± 108 | 217 ± 95 | 0.46 |
| Serum creatinine (mg/dL) | 1.2 ± 1 | 1.6 ± 2.7 | 0.19 |
| Serum sodium (meq/L) | 132 ± 6.9 | 134 ± 6.4 | 0.1 |
| Serum potassium (meq/L) | 4.5 ± 0.9 | 4.5 ± 1.2 | 0.69 |

Data are expressed as mean ± standard deviation for continuous variables, and number (percentage) for categorical variables.
